# The non-modifiable factors age, gender, and genetics influence resistance exercise

**DOI:** 10.3389/fragi.2022.1005848

**Published:** 2022-09-12

**Authors:** Claudio Viecelli, Collin Y. Ewald

**Affiliations:** ^1^ Institute of Molecular Systems Biology, ETH Zurich, Zurich, Switzerland; ^2^ Institute of Translational Medicine, Department of Health Sciences and Technology, ETH Zürich, Schwerzenbach, Switzerland

**Keywords:** resistance exercise, age, gender, genetics, modifier

## Abstract

Muscle mass and force are key for movement, life quality, and health. It is well established that resistance exercise is a potent anabolic stimulus increasing muscle mass and force. The response of a physiological system to resistance exercise is composed of non-modifiable (*i.e.,* age, gender, genetics) and modifiable factors (*i.e.,* exercise, nutrition, training status, etc.). Both factors are integrated by systemic responses (*i.e.,* molecular signaling, genetic responses, protein metabolism, etc.), consequently resulting in functional and physiological adaptations. Herein, we discuss the influence of non-modifiable factors on resistance exercise: age, gender, and genetics. A solid understanding of the role of non-modifiable factors might help to adjust training regimes towards optimal muscle mass maintenance and health.

## Introduction

The importance of muscle mass and strength and their associated metabolic functions in the performance of exercise and activities in daily living is recognized as a critical factor in life (Fuhrman et al., 1951; Kotler et al., 1989; Wolfe, 2006). Skeletal muscle is a highly plastic tissue consistently adapting to different physiological conditions, such as mechanical loading (Goldberg et al., 1975; Fry, 2004; Rindom et al., 2019) or metabolic stress (ROONEY et al., 1994; Carey Smith and Rutherford, 1995; Schott et al., 1995), disuse (Bodine, 2013), hypoxia (Hoppeler et al., 2008), weightlessness (Desplanches, 1997), cold exposure (Buser et al., 1982; van den Berg et al., 2011), and nutritional modifications (Vogt et al., 2003). As such, it adapts to physical activity (exercise) or inactivity (disuse, disease, injury) (Flück and Hoppeler, 2003).

Physical activity, the movement of the human body by skeletal muscles that expends energy, was evolutionary advantageous as it allowed for traveling and discovering new habitats (Lieberman, 2013). The evolution of exercise coincides with the evolution of hunting and gathering, as foraging for food increased physical activity significantly (Lieberman, 2015). The absence of the need for daily hunting and gathering for food or water resulted in inactivity in our more comfortable lifestyles today. In order to counteract this inactivity, we have to engage in voluntary physical activity that is planned, structured, repetitive, and undertaken to sustain or improve health and fitness, defined as exercise (Lieberman, 2021). Exercise challenges whole-body homeostasis, demanding an orchestrated systemic response permitting to equilibrate metabolic demands of contracting skeletal muscles (Hawley et al., 2014). Hence, the resulting metabolic and morphological adaptations are highly exercise-specific.

The combinatorial possibilities of intensity and duration allow for a plethora of exercise types. Two of the most extensively studied types of exercise are endurance and resistance exercise.

Endurance exercise is typically characterized by continuous bouts of lower-intensity contractions (Coffey and Hawley, 2017) allowing the individual to sustain exercise for a prolonged time. Typical endurance training is, for instance, walking, running, cycling, and swimming. Prolonged contractile activity at a lower intensity denotes a challenge to the metabolic system disrupting intracellular concentrations of oxygen, lactate, reactive oxygen species, adenosine triphosphate, nicotinamide adenine dinucleotide, and calcium (Coffey and Hawley, 2007). These disruptions initiate signaling cascades converging on peroxisome-proliferator-activated receptor gamma coactivator 1 alpha regulating mitochondrial biogenesis (Baar et al., 2002; Wu et al., 2002; Pilegaard et al., 2003), capillarity (Saltin and Gollnick, 1983), and substrate utilization (Holloszy and Coyle, 1984). Therefore, endurance exercise is associated with adaptations to increase oxidative capacity (Hawley, 2002).

In contrast to endurance exercise, resistance exercise (RE) is associated with short duration and higher to maximal intensity contractions (Egan and Zierath, 2013). RE challenges the mechanical integrity (Ingber, 2003a; Ingber, 2003b) and metabolic homeostasis of muscles (Goto et al., 2005; Schoenfeld, 2013). The classical morphological and neural adaptions to RE include for instance changes in muscle fiber size (McDonagh and Davies, 1984; Jones et al., 1989) and architecture (Franchi et al., 2017), myofibrillar growth and mitochondrial proliferation (Macdougall et al., 1979; MacDougall et al., 1980), metabolic profile (Zanuso et al., 2017), tendon stiffness and thickness (Reeves et al., 2003; Kongsgaard et al., 2007), firing frequency (Leong et al., 1999), cortical adaptations (Perez et al., 2004), spinal reflexes (Aagaard et al., 2002) and antagonist coactivation (Baratta et al., 1988). In addition, cardiovascular improvements are reported, such as enhanced blood pressure control (MacDonald et al., 2016), improved insulin sensitivity controlling blood glucose (Codella et al., 2018), and weight management (Paoli et al., 2015). The high plasticity of skeletal muscle is retained lifelong as RE increases muscle mass (DeLorme, 1945; Phillips, 2014) in men and women of all ages (Westcott et al., 2009).

Conceptually, the response of a physiological system to RE comprises non-modifiable (*i.e.,* age, gender, genetics) and modifiable factors (*i.e.,* type and duration of exercise, nutrition, training status, etc.) (Spiering et al., 2008). Both factors are integrated by systemic responses (*i.e.,* molecular signaling, genetic responses, protein metabolism, etc*.*), consequently resulting in functional adaptations (Spiering et al., 2008). While the contribution of RE descriptors has been reviewed elsewhere (Viecelli and Aguayo, 2022), the aim of this review is to discuss the influence of age, gender, and genetics on RE outcomes.

## Age

### Age-associated changes on the cellular and molecular level

Aging, a decline in physiological function, is universal and impacts quality of life (Selman et al., 2012; Lemaître et al., 2015). In contrast to chronological aging, whereby aging is referred to as a function of time an individual existed, biological aging is referred to epigenetic changes and expresseses how fast the cellular machinery deteriorates, depending on the individual genetic setup and lifestyle factors, such as nutrition and exercise (Sillanpää et al., 2019). As biological aging impacts musculoskeletal health, in this review, aging is referred to as biological aging.

**FIGURE 1:**
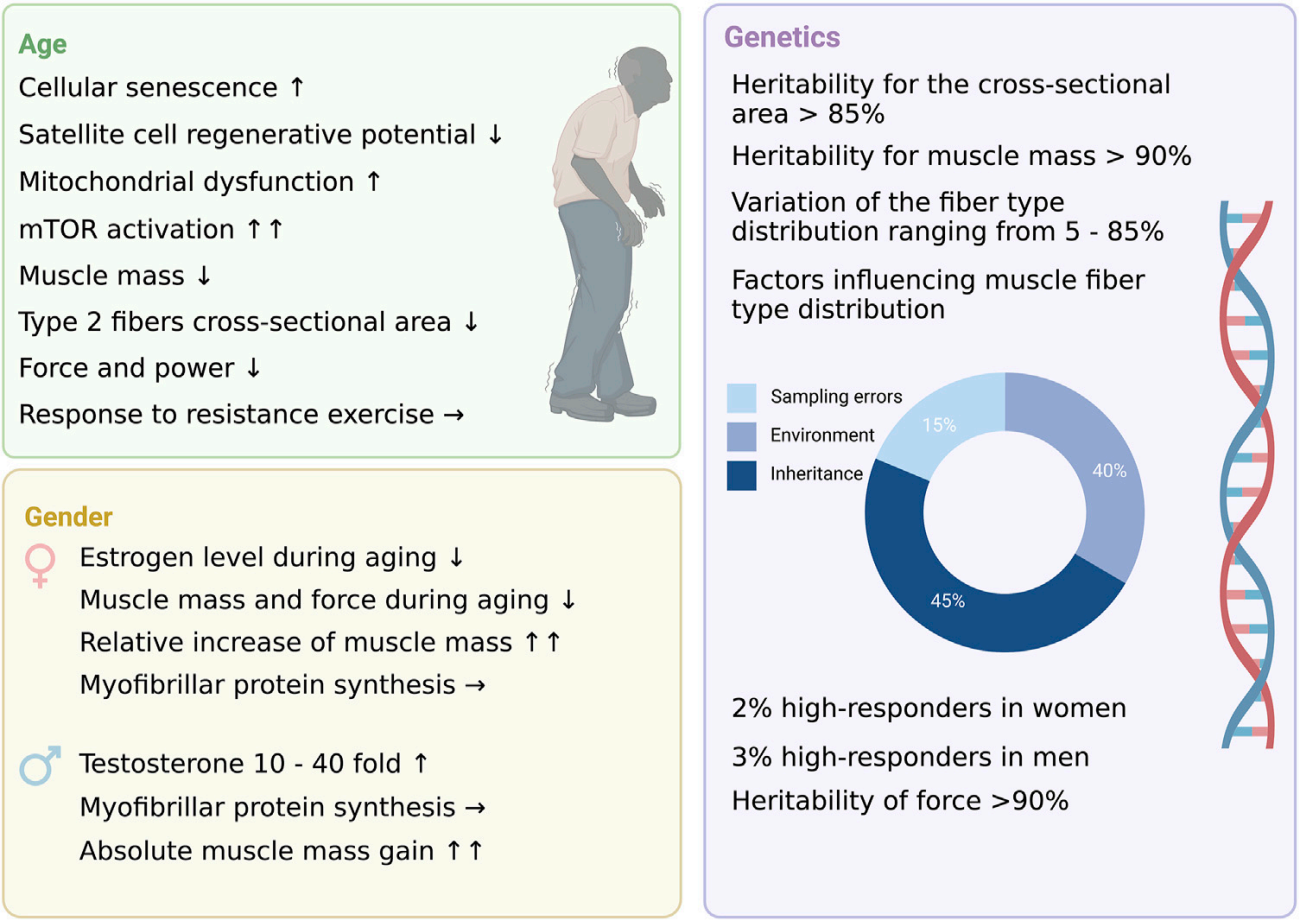
The three non-modifiable factors of resistance training.

On the cellular level, aging is associated with the occurrence, accumulation, and consequences of molecular damage (Rattan, 2016), resulting from different sources (*i.e.,* reactive oxygen species (ROS), free radicals and their associated biochemical interactions, spontaneous DNA duplication, translational, posttranslational errors, etc*.*) (Rattan, 2009). These interactions, as observed in cell cultures of human diploid cell strains, contribute to the finite replicative capacity of cells (Hayflick, 1965), ultimately resulting in proliferative cell cycle arrest attributed to telomere shortening (Harley et al., 1990; Bodnar et al., 1998). These processes gave rise to the concept of cellular senescence. It is noteworthy that chronic activation of tumor suppressors (*i.e.,* retinoblastoma protein and the transcription factor p53) has also been shown to induce cell cycle arrest (Harvey and Levine, 1991; Serrano et al., 1997). Aging might therefore be a function of the progressive accumulation of senescent cells over a lifetime, consequently associated with a disruption in tissue homeostasis and integrity, reducing responses to physiological stressors (Sharpless and DePinho, 2007; Signer and Morrison, 2013; Van Deursen, 2014a; Muñoz-Espín and Serrano, 2014) (Figure 1).

Moreover, cellular senescence changes the cellular protein expression and secretion; the latter is termed secretome (Agrawal et al., 2010). This impacts complex biological processes used for development, tissue repair, and age-related diseases (Van Deursen, 2014b). The secretome of senescent cells, consisting of cytokines, proteases, chemokines, growth factors, and extracellular vesicles, can either be beneficial or detrimental depending on the composition and senescence-inducing stressors (Coppé et al., 2008). The 2 – 4 fold elevated serum levels of secreted TNFα, IL-6, and C-reactive protein (CRP) compared to younger individuals promote inflammation that can accelerate aging (Schaap et al., 2006; Bian et al., 2017; Can et al., 2017; Marzetti et al., 2019).

In addition, multiple signaling pathways such as Wnt (Du et al., 2014; Hu et al., 2014), TGFβ (Carlson et al., 2008) and FGF (Bernet et al., 2014) promote cell-cycle inhibitors and, thus, contribute to cellular senescence in skeletal cells.

In aging skeletal muscle, the proliferative potential and the number of satellite cells decline significantly (Boldrin et al., 2010; García-Prat et al., 2013). The observation that in elderly people, the proportion and the cross-sectional area (CSA) of type II fibers are significantly reduced, and these fibers showed a lower satellite cell content led to the conclusion that satellite cell dysfunction could be a driver for muscle aging (Verdijk et al., 2007). However, this is still controversial as satellite cell depletion in adult mice did not affect muscle atrophy (Jackson et al., 2012; Fry et al., 2015), suggesting that satellite cells are only crucial in regenerative processes and do not support size maintenance of aged muscle fibers (Fry et al., 2015; Murach et al., 2018). The secretome might influence extrinsic factors from satellite cell niches, such as FGF (Pawlikowski et al., 2017), TGFβ (Carlson et al., 2009), and myostatin (McKay et al., 2012), negatively affecting satellite regenerative potential (Bentzinger et al., 2010).

Emerging evidence suggests a key role of mitochondria in aging and age-associated diseases (Amorim et al., 2022). Caloric restriction has been shown to extend lifespan in yeast (Lin et al., 2002), *Caenorhabditis elegans* (Schulz et al., 2007), and mammals (Nisoli et al., 2005), indicating a role of mitochondria as longevity signaling pathways converge, *inter alia*, on mitochondrial regulation (López-Lluch and Navas, 2016). By contrast, the disruption of mitochondrial function is observed in senescent cells (Yoon et al., 2003; Byun et al., 2012; Lafargue et al., 2017). Age-associated mitochondrial damage increases ROS accumulation and leads to energy deficiency in skeletal muscle (Lourenço dos Santos et al., 2015; Kadoguchi et al., 2020), rendering skeletal muscle susceptible to atrophy mediated by FoxO-dependent autophagy (Tezze et al., 2017). Hence, failure in mitochondrial dynamics has a negative impact on muscle function and maintenance (Favaro et al., 2019; Huang et al., 2019) and this mitochondrial dysfunction contributes to a proteolytic shift (Mankhong et al., 2020).

In addition, the mechanistic target of rapamycin (mTOR) has been found to be hyperactivated during aging, resulting in mitochondrial dysfunction and increased oxidative stress (Joseph et al., 2019; Tang et al., 2019), ultimately leading to fiber decay (Vainshtein and Sandri, 2020) (Figure 1). The reason for this hyperactivation has not yet been elucidated, and due to the complexity and interrelatedness, the molecular mechanisms of muscular aging are not fully understood (Cruz-Jentoft and Sayer, 2019). Nevertheless, it is undebated that aging interferes with skeletal muscle homeostasis resulting in an imbalance of protein synthesis and degradation, promoting proteolytic signaling pathways (Cruz-Jentoft and Sayer, 2019).

### Aging and the force-generating capacity

Human aging is associated with a reduced force-generating capacity attributed to multiple changes such as the loss of muscle mass (Janssen et al., 2000a; Williams et al., 2002; Topinková, 2008; Degens and Korhonen, 2012; Mitchell et al., 2012), fiber type shifting (Andersen, 2003; Verdijk et al., 2007; Barnouin et al., 2017), muscle architecture and ultrastructure (Binzoni et al., 2001; Kubo et al., 2003; Morse et al., 2005), and neural control (Urbanchek et al., 2001; Morse et al., 2004), significantly impacting the health of elderly (Rosenberg, 1989; Baumgartner et al., 1998; Cruz-Jentoft et al., 2010; Karaguzel and Holick, 2010; Levinger et al., 2016; Lo et al., 2017).

Unfortunately, skeletal muscle cannot escape the aging process and, hence, deteriorates as a function of time (Degens and Korhonen, 2012). While post-puberty and during adulthood, muscle mass and strength are stable in healthy individuals, starting between the 4th and 5th decade of life, atrophic processes are favored, resulting in a decrease in muscle mass and strength (Williams et al., 2002). A recent quantitative review calculated the median loss of muscle mass per decade in men as 4.7 and 3.7% in women, respectively (Mitchell et al., 2012). By the age of 80 years, 30% of the peak muscle mass of an individual is lost because of aging (Janssen et al., 2000a; Topinková, 2008). Muscle mass loss is not distributed uniformly over the whole body, as determined in a magnetic resonance imaging study of 200 women and 268 men, whereby the rate of loss of muscle mass in lower limb muscles was more than double in comparison to upper limb muscles (Janssen et al., 2000b). Although men possess more muscle mass than women, muscle mass loss is similar between sexes when the loss is regarded as a proportion of peak muscle mass (Janssen et al., 2000b; Silva et al., 2010). Hence, there is no evidence of sexual dimorphism in the age-associated loss of muscle mass (Runge et al., 2004; Maden-Wilkinson et al., 2015). As men do have 1.5–2 times larger muscle mass and strength than women, they are reaching the disability threshold later in life (∼1.5 years) (Miller et al., 1993; Goodpaster et al., 2001).

Aging is associated with a reduction predominantly in the cross-sectional area (CSA) of type II fibers (Barnouin et al., 2017). Hence, the proportion and volume of type I fibers increase (Andersen, 2003; Verdijk et al., 2007; Barnouin et al., 2017). Between the age of 22 and 74, a reduction of type II CSA from 58 to 52% was observed for the *m. vastus lateralis* in men (Barnouin et al., 2017). Given that the mechanical tension of type II fibers is 1.4 times higher than the specific tension of type I fibers (Bottinelli et al., 1996; Widrick et al., 1996), at best, it could explain a 2% and not a 45% force reduction that was reported between these ages (Degens et al., 2009). It is also noteworthy that there are studies not observing tensional differences between fiber types (Ottenheijm et al., 2005; Degens and Larsson, 2007; Meijer et al., 2015). Hence, it is fair to conclude that fiber type shifting only minimally accounts for the age-associated force reduction seen during aging.

Muscle architecture and ultrastructure change during aging. While aging or detraining decreases the pennation angle of the fascicle (Binzoni et al., 2001; Kubo et al., 2003; Morse et al., 2005) when muscle mass is lost (Janssen et al., 2000a; Williams et al., 2002; Topinková, 2008; Degens and Korhonen, 2012; Mitchell et al., 2012), vice versa RE increases the fascicle pennation angle due to the optimization of the packaging of large fibers between the aponeurosis (Aagaard et al., 2001; Blazevich, 2006; Seynnes et al., 2007; Bloomquist et al., 2013). A decrease in the pennation angle increases the force and power-generating capacity because of an enhancement of the cosine function. Therefore, the change in muscle architecture (*i.e.,* a decrease in pennation angle of fascicle) attenuates the loss of force and power (Degens et al., 2009).

Aging has been associated with ultrastructural changes such as increases in connective tissue and fat infiltration (Goodpaster et al., 2001; Degens and McPhee, 2013; Power et al., 2014) as extensively researched using ultrasound (Pillen et al., 2009; Akima et al., 2017). The non-contractile area was found to be twice the size when comparing young versus old men (*p <* 0.05) and could thus explain the observed force loss better than the reduction of type II CSA (Power et al., 2014).

Reduction of force in the elderly may also be attributed to the comprised ability to recruit the muscle voluntarily (Morse et al., 2004). In rat muscles, the denervation of muscle fibers explained 11% of force reduction (Urbanchek et al., 2001). Furthermore, increased co-activation of antagonist muscles has been shown to interfere with maximum force production in the elderly attenuating specific tension (Morse et al., 2004).

Given that skeletal muscle mass accounts for up to 40% of an individual total body mass (Frontera and Ochala, 2015), the loss of muscle mass and strength has a fundamental impact on health in the elderly population as it is associated with the risk of adverse outcomes such as physical disability, poor quality of life and death (Rosenberg, 1989; Baumgartner et al., 1998; Cruz-Jentoft et al., 2010). Moreover, the close link between skeletal muscle mass and bone mineral density leads to bone loss when skeletal muscle mass deteriorates. Osteopenia, the loss of bone mass (Karaguzel and Holick, 2010), together with sarcopenia, present major clinical problems. The impairment of locomotory functions leads to comprised balance and increases the risk of falls promoting osteoporotic fractures (Levinger et al., 2016). Hence, low skeletal muscle mass is a driver of public medical costs as hospitalization within this cohort has a high prevalence (Lo et al., 2017). In the United States alone, the total cost of hospitalizations in individuals with sarcopenia was estimated to be $40.4 billion in 2014 (Goates et al., 2019). In Switzerland, a quarter of the elderly was affected by sarcopenia in 2016 (Wearing et al., 2020).

### Aging and resistance exercise

RE is a potent anabolic countermeasure to fight sarcopenia as it increases muscle mass and strength even in geriatric individuals. Fiatarone and colleagues (Fiatarone et al., 1990) subjected ten frail, institutionalized volunteers aged 90 ± 1 year to 8 weeks of high-intensity training. Strength gains averaged 174 ± 31% (mean ± SEM) in the 9 subjects who completed the training. Midthigh muscle area increased 9 ± 4.5%. Therefore, resistance exercise leads to significant gains in muscle strength, size, and functional mobility among frail residents of nursing homes.

Churchward-Venne et al. (2015), investigated the prevalence of non-responders to RE, assessing lean body mass (LBM), muscle fiber size, strength, and/or physical function after 12 (*n* = 110) and 24 (*n* = 85) weeks of RE. In response to resistance exercise training, LBM increased by 0.9 ± 0.1 kg (range: −3.3 to +5.4 kg; *p* < 0.001) from 0 to 12 weeks of training and by 1.1 ± 0.2 kg (range: −1.8 to +9.2 kg; *p* < 0.001) from 0 to 24 weeks. Moreover, muscle fiber analysis showed an average increase of type 1 and 2 muscle fiber size by 324 ± 137 mm^2^ (range: −4,458 to +3,386 mm^2^; *p* = 0.021) and 701 ± 137 mm^2^ (range: −4,041 to +3,904 mm^2^; *p* < 0.001) from 0 to 12 weeks for type 1 and 2 muscle fiber respectively. From 0 to 24 weeks, type 1 and 2 muscle fiber size increased by 360 ± 157 mm^2^ (range: −3,531 to +3,426 mm^2^; *p* = 0.026) and 779 ± 161 mm^2^ (range: −2,728 to +3,815 mm^2^; *p* < 0.001) for type 1 and 2 muscle fiber, respectively. Functional assessment for the 1-RM strength on the leg press and leg extension showed an increase by 33 + 2 kg (range: −36 to +87 kg; *p* < 0.001) and 20 + 1 kg (range: −22 to +56 kg; *p* < 0.001) from 0 to 12 weeks and an increase by 50 + 3 kg (range: −28 to +145 kg; *p* < 0.001), and 29 + 2 kg (range: −19 to +60 kg; *p* < 0.001) from 0 to 24 weeks for the leg press and leg extension 1-RM respectively. Lastly, further functional assessments such as chair-rise time decreased by 1.3 + 0.4 s (range: +21.6 to −12.5 s; *p* = 0.003) from 0 to 12 weeks and decreased by 2.3 + 0.4 s (range: +10.5 to −23.0 s; *p* < 0.001) from 0 to 24 weeks.

The authors observed that in all subjects, at least one positive interventional outcome was found and concluded that there is no evidence for non-responders to RE in their study.

Wroblewski et al. (2011) examined body composition, peak torque, and magnetic resonance imaging (MRI) of bilateral *quadriceps* of 40 highly trained individuals aged 40–81 years. MRI quantification of mid-thigh muscle area (*p* = 0.31) and lean mass (*p* = 0.15) revealed no increase with age, and a significant relationship of retention of mid-thigh muscle area (*p* > 0.0001) was observed. Additionally, in these highly trained individuals, specific strength (strength per *quadriceps* area) did not significantly decline as a function of aging (*p* = 0.06). Therefore, the authors concluded that aging alone could not explain the commonly observed decline in muscle mass and strength, and chronic disuse might be a stronger driver of atrophy rather than aging.

Although a large heterogeneity of hypertrophy in response to RE is observed, chronic RE is associated with increases with positive effects, as observed by (Churchward-Venne et al., 2015). It is again pointing to a necessity of a lifelong intervention. As such, muscle plasticity is not compromised due to the aging process *per se*. Hence, we strongly encourage even the oldest olds to implement regular resistance exercise into their daily habit.

## Gender

Besides the reproductive organs, before puberty, no significant anthropometrical differences between boys and girls exist. Sexual dimorphism is pronounced as puberty begins due to hormonal changes.

Testosterone serum level, for example*,* is 10- to 40-fold higher in men at rest (Kraemer et al., 1991; Vingren et al., 2010) and because of its androgenic-anabolic potential (Brodsky et al., 1996; Bhasin et al., 1997; Snyder et al., 2000) thought to mediate muscle mass through the ability to increase (Urban et al., 1995) the synthesis and/or decreasing (Zhao et al., 2008) the breakdown of myofibrillar protein. Estrogen is thought to regulate the muscle mass of women as this hormone exerts the capacity to downregulate myofibrillar protein breakdown (Pollanen et al., 2007). Estrogen receptors have been found in skeletal muscle tissue, tendons, and ligaments and are thought to regulate skeletal muscle proteins and enhance the sensitivity to anabolic stimuli (Hansen and Kjaer, 2014). During aging, the estrogen levels decrease, affecting women detrimentally as they experience a rapid decline in muscle mass and force (Hansen and Kjaer, 2014). Postmenopausal hormone replacement therapy reversed these changes by an increase in myogenic gene expression, indicating the role in muscle anabolism (Dieli-Conwright et al., 2009).

The menstrual cycle was the subject of multiple studies examining muscle strength, whereby little or no differences were found during the different stages of the cycle (Elliott et al., 2003; Fridén et al., 2003; Bambaeichi et al., 2004). However, as many factors can influence exercise performance, this topic warrants more research.

As such, men and women are capable of increasing muscle mass and strength in response to RE (Abe et al., 2000; Hubal et al., 2005; Kosek et al., 2006). However, it must be understood that women start with less muscle mass, thus biasing, relative changes of muscle mass increase in their favor.

### Gender differences in resistance exercise

It is well established that RE provides a potent anabolic stimulus for both sexes, mediated partly by acute and chronic and hormonal changes, including testosterone, insulin-like growth factor 1 (IGF-1), growth hormone (GH), and dehydroepiandrosterone sulfate (DHEA-S) (Consitt et al., 2002; Kahn et al., 2002; Kraemer and Ratamess, 2005; Fleck and Kraemer, 2014). However, RE-induced changes differ significantly between women and men (Figure 1).

While serum testosterone levels following heavy RE are acutely elevated in men (Fleck and Kraemer, 2014) they do not change in women after RE (Kraemer et al., 1991; Kraemer et al., 1993; Staron et al., 1994; Hakkinen and Pakarinen, 1995). For GH, the response to RE seems to be similar between gender, as RE induced a post-exercise increase of GH in women and men (Kraemer et al., 1991; Hakkinen and Pakarinen, 1995). While research on the acute response of IGF-1 to RE is equivocal (Kraemer et al., 1991; Kraemer et al., 1993; Consitt et al., 2001; Kraemer and Ratamess, 2005), the combination of GH and IGF-1 seems to play a testosterone-compensatory effect in women (Kraemer et al., 2010) as women show a markedly increase in fiber CSA as a result of regimented RE (Staron et al., 1994) despite low levels of testosterone.

DHEA-S is a peripheral precursor in testosterone metabolism (Yamazaki and Shimada, 1997), accounting for roughly 90% of circulating testosterone in women (Baulieu, 1996; Labrie et al., 1997) being the predominant adrenal steroid hormone in women and men (Nakamura and Aizawa, 2017). An acute bout of RE increased blood DHEA-S levels in women and men (Riechman et al., 2004) while 8 weeks of RE significantly increased resting DHEA-S levels in women (Aizawa et al., 2003). In addition, Aizawa *et al.* (Aizawa et al., 2006) reported that DHEA-S levels positively correlated with leg extensor power in women (*p < 0.001*) but not in males. As such, DHEA-S levels might be an important driver of strength development in female athletes. Thus, gender differences in resting anabolic hormone levels and responses to exercise do exist.

Multiple studies addressed gender differences in RE-induced hypertrophy and force. Roth *et al.* (Roth et al., 2001) examined the possible influence of age and gender on muscle volume responses to strength training. Eight young men, six young women, nine older men, and ten older women underwent a 6-months whole-body strength training program that exercised all major muscle groups of the upper and lower body 3 days per week. The authors used MRI to assess thigh and *quadriceps* muscle volume and mid-thigh CSA before and after the interventional period. Muscle volume increased significantly in all age and gender groups in response to strength training (*p* < 0.001). No statistically significant difference between the groups was found. Neither gender nor age influenced the muscle volume response to strength training.

In a study examining the effects of age, gender, and myostatin genotype on the hypertrophy response to heavy resistance strength training (Ivey et al., 2000), recruited 11 young men (25 ± 3 years) and women (26 ± 2 years), 12 older men (69 ± 3 years), and 11 older women (68 ± 2 years). The participants underwent 9 weeks of resistance exercise, consisting of knee extensions of the dominant leg three times per week. Bilateral muscle volume was measured using MRI before and after the intervention. Absolute increases in muscle volume were greater in men than in women (204 ± 20 vs*.* 101 ± 13 cm^3,^ *p < 0.01*). Even after adjusting for baseline muscle volume, a gender effect remained. Additionally, 31 weeks of detraining showed a significantly greater loss of absolute muscle volume in men than in women (151 ± 13 vs*.* 88 ± 7 cm^3,^ *p < 0.05*). The authors concluded that muscle mass response is affected by gender as men increased their muscle volume about twice as much as women.

In line, Bamman and colleagues (Bamman et al., 2003) examined gender differences in resistance-training-induced myofiber among older adults and recruited nine older men (69 ± 2 years) and five older women (66 ± 1 year). Using biopsies of the *vastus lateralis* to determine CSA after 26 weeks of resistance exercise three times a week, including knee extension, leg presses, or squats. In addition, 1-RM was assessed pre- and post-intervention. Although the intervention increased CSA for all fiber types (*i.e.,* I, IIA, IIX) in both sexes, hypertrophy (*p < 0.05*) and strength gains (*p < 0.05*) were greater in men when compared with women.

Walts and colleagues (Walts et al., 2008) investigated the influence of sex and race on the effects of strength training on thigh muscle volume. They recruited 181 inactive healthy women (63 ± 0.9 years, *n* = 99) and men (63 ± 0.9 years, n = 82) who were subjected to unilateral knee extension of the dominant leg trice a week for 10 weeks. *Quadriceps* muscle volume was measured using computed tomography before and after the intervention period. Absolute increases in muscle volume were significantly greater (*p < 0.001*) in men than in women, although both sex groups increased muscle volume significantly (*p < 0.001*) as a response to strength training.

Hubal et al. (2005), tested 342 women and 243 men. The participants were subjected to isometric and dynamic strength training regimes of the *biceps brachii* of the non-dominant arm. MRI was used to determine the CSA before and after 12 weeks of progressive, dynamic resistance training. Men experienced 2.5% greater absolute gains for the muscle CSA (*p* < 0.01) when compared to women. However, despite absolute gain, relative increases in strength measures, *i.e.,* maximal voluntary isometric contraction and 1 repetition maximum (1RM), were greater in women versus men (*p* < 0.05).

These results are in line with a study conducted by (West et al., 2012) that conducted a sex-based comparison of myofibrillar protein synthesis (MPS) after a single bout of high-intensity RE in the fed state of eight men and eight women. Participants underwent constant infusions of L-[*ring*-^13^C_6_] phenylalanine on consecutive days with serial muscle biopsies. Results showed that although serum testosterone increased 45-fold in men compared to women, MPS did not differ between men and women when comparing 1–5 h post-exercise and after protein ingestion following 24 h recovery. Although testosterone levels are significantly different in men and women in response to a single bout of high-intensity RE, MPS is robustly elevated in both sexes. Hence, there seems to be a disassociation from post-exercise testosterone levels and MPS as MPS is elevated in women, exerting low systemic testosterone concentrations.

Although men and women differ in their basal anabolic hormone levels and thus also in responses to exercise, women and men display similar changes in muscle mass and force as a function of RE (Figure 1). However, absolute increases in muscle mass are greater in men than in women. Nevertheless, MPS is robustly elevated in both sexes as a response to RE.

Therefore, it should be strongly advocated for all and especially encourage women to perform resistance exercise training.

## Genetics

Genetic factors influence phenotype traits. This is also the case for traits related to sports performance (MacArthur and North, 2005) and might explain why certain individuals do have the genetic makeup to become elite bodybuilders, for example. Up to the year 2008, over 200 autosomal genes and 18 mitochondrial genes were associated with improving fitness and performance (Bray et al., 2009). Sports performance, however, is influenced by far more than one gene and is considered a highly complex, polygenic trait (Pickering et al., 2019). Furthermore, even identical twins with the same genetic machinery do have subtle distinct physical and personality traits (McArdle et al., 2010). Thomis et al. (1998) studied maximal static, eccentric torques, and arm components estimated by anthropometry and measured by computed tomography in 25 monozygotic and 16 dizygotic twins (22.4 ± 3.7 years). They reported a heritability for the arm CSA measurements of more than 85% without the significance of common environmental factors (Thomis et al., 1998). Huygens and colleagues (Huygens et al., 2004) estimated the genetic and environmental contribution to the variation in skeletal muscle mass and strength in 748 sibling pairs of young brothers (24.3 ± 4.5 years) and an additional 25 monozygotic and 15 dizygotic male twins from the Twin & Training Study by (Thomis et al., 1998). They reported transmissibility for muscle mass to be greater than 90%. In regard to the genetic influence on strength, Thomis and colleagues (MA et al., 1998) quantified strength after 10-week high-resistance training in 25 monozygotic and 16 dizygotic twins (22.4 ± 3.7 years) and reported a heritability for 1 RM strength of 77% for the elbow flexor. This is lower than what Huygens *et al.* found in 748 sibling pairs of young brothers (24.3 ± 4.5 years) for the elbow flexors, where they reported a heritability of >90% (Huygens et al., 2004). Thus, for twins, the inheritance of muscle mass and strength is extremely high, between 85 and 90%.

By contrast, examining to what extent human skeletal muscle fiber type proportion is under the control of genetic factors by analyzing *vastus lateralis* muscle biopsies from 32 pairs of brothers, 26 pairs of male and female dizygotic twins, and 35 pairs of male and female monozygotic twins (Simoneau and Bouchard, 1995). They found that roughly 45% of the variance is associated with inherited factors, 40% are environmental influences, and 15% are attributed to sampling errors (Simoneau and Bouchard, 1995).

Given the difference in the genetic setup of the general human population, it should then not come as a surprise that Hubal and colleagues (Hubal et al., 2005) demonstrated highly dissimilar responses to 12 weeks of resistance training of the elbow flexors in men and women, whereas CSA change ranged from −2.3 to 59% in women and −2.5–55.5% in men. This great variance in the CSA change led to the notation of low responding (0.08% of men and women) and high responding (3% of men and 2% of women) individuals.

In a retrospective study assessing the prevalence of unresponsiveness of older men and women to augment muscle mass and strength, (Churchward-Venne et al., 2015) examined the adaptive response to 12 and 24 weeks of supervised resistance-type exercise training in older (>65 years) men and women. The 24-weeks training intervention consisted of evaluation at 12 and 24 weeks. It was observed that the duration of resistance training is an important factor as there were individuals who demonstrated little to no effect after 12 weeks of training but substantial improvements after 24 weeks of training.

In a study quantifying high- and low-responders by resistance training-induced changes in muscle size and strength, data of untrained healthy men and women (age 19–78 years, *n* = 287 with 72 controls) were examined by (Ahtiainen et al., 2016). Resistance training-induced muscle size changed from −11 to 30%, and strength changed from −8 to 60% in men and women. Interestingly, looking at the correlated data of changes in muscle size and muscle strength, some individuals experienced a resistance exercise-induced decrease in muscle size (*ca.* −10%) but a substantial increase in 1-RM (*ca.* 28%). This might be due to better innervation of the muscle leading to increased strength.

Pérusse et al. (1987) used path analysis to assess inherited and environmental variance components in physical fitness indicators measured in 1,630 subjects from 375 families and assessed muscular strength, among other things. The used BETA model allowed the partition of transmissible variance, defined as factors transmitted from parents to offspring, into genetic factors and cultural components. The results indicated that the transmissible variance accounted for 63% of muscular strength, while genetic factors alone were found to account for 30% of the muscular strength of the phenotypic variance. Concerning the phenotypic variation observed in muscular strength, cultural inheritance was reported to account for 31% and environmental factors for 37%.

In summary, phenotypic variation in force and muscle mass seems to be influenced by genetic and environmental factors (Figure 1). However, the contribution of heredity is moderate in non-identical twins, and non-transmissible environmental factors are the drivers of muscle mass and strength. Additionally, it must be recognized that the terms non-, low- and high-responders have to be treated with caution (Figure 1). Adaptations to resistance training are multifaceted and associated with health benefits. Focusing only on a small number of variables and determining the level of responders is too narrow-minded. Besides that, non-responsiveness is extremely unlikely. None of the mentioned studies followed up within the non-responsive cohorts by adjusting the training variables (*e.g.,* volume, frequency, duration, *etc.*).

## Conclusion

The non-modifiable factors age, gender, and genetics influence muscle mass and force as a function of RE. Although aging is associated with a reduced force-generating capacity attributed to multiple changes such as the loss of muscle mass, fiber type shifting, muscle architecture, and ultrastructure and neural control that can impact the health of the elderly, the plasticity of muscle is retained lifelong. Hence, vigorous RE can reverse or attenuate age-associated loss of muscle mass and strength.

Gender also influences muscle mass and strength. Men and women display similar changes in muscle mass and force as a function of RE. Although muscle mass increases are greater in men than women, MPS is robustly elevated post-exercise in both sexes. Therefore, men and women are encouraged to implement RE as a weekly routine to maintain and increase muscle mass and strength.

As stated above, the phenotypic variation in muscle mass and force seems to be influenced by genetic and environmental factors. However, the contribution of heredity is moderate, and non-transmissible environmental factors are the drivers of muscle mass and force. Hence, vigorous RE contributes to a greater extent to muscle mass and force.

In this review, we discussed the effects of the non-modifiable factors age, gender, and genetics separately. Future research examining the interactional or combined effects of those factors could further contribute to the understanding of the non-modifiable factors of RE.

A schematic representation of the key concepts for age, gender, and genetics are summarized in this review.
